# Crystal Structure of BamB from *Pseudomonas aeruginosa* and Functional Evaluation of Its Conserved Structural Features

**DOI:** 10.1371/journal.pone.0049749

**Published:** 2012-11-26

**Authors:** Katarina Bartoš Jansen, Susan Lynn Baker, Marcelo Carlos Sousa

**Affiliations:** Department of Chemistry and Biochemistry, University of Colorado at Boulder, Boulder, Colorado, United States of America; Dundee University, United Kingdom

## Abstract

The assembly of β-barrel Outer Membrane Proteins (OMPs) in the outer membrane is essential for Gram-negative bacteria. The process requires the β-Barrel Assembly Machine (BAM), a multiprotein complex that, in *E. coli*, is composed of the OMP BamA and four lipoproteins BamB-E. Whereas BamA and BamD are essential, deletion of BamB, C or E produce membrane permeability defects. Here we present the high-resolution structure of BamB from *Pseudomonas aeruginosa*. This protein can complement the deletion of *bamB* in *E. coli* indicating that they are functionally equivalent. Conserved structural features include an eight-bladed β-propeller fold stabilized by tryptophan docking motifs with a central pore about 8 Å in diameter at the narrowest point. This pore distinguishes BamB from related β-propellers, such as quinoprotein dehydrogenases. However, a double mutation designed to block this pore was fully functional indicating that the opening is not essential. Two loops protruding from the bottom of the propeller are conserved and mediate binding to BamA. Conversely, an additional loop only present in *E. coli* BamB is not required for function. A cluster of highly conserved residues in a groove between blades 6 and 7 is crucial for proper BamB folding or biogenesis. It has been proposed that BamB may bind nascent OMPs by β-augmentation to its propeller outer strands, or recognize the aromatic residue signature at the C-terminus of OMPs. However, Isothermal Titration Calorimetry experiments and structural analysis do not support these proposals. The structural and mutagenesis analysis suggests that the main function of BamB is to bind and modulate BamA, rather than directly interact with nascent OMPs.

## Introduction

The outer membrane of Gram-negative bacteria is a unique structure that is almost impermeable to hydrophilic molecules and allows only slow diffusion of hydrophobic ones [1]. Integral Outer Membrane Proteins (OMPs) fold into characteristic β-barrels that span the outer membrane [2] and are often selective channels or porins that allow exchange of nutrients and waste products between the cell and the environment [1]. Folding and insertion of OMPs into the outer membrane is an essential process for Gram-negative bacteria [3]. OMPs are synthesized in the cytosol and translocated across the inner membrane by the SecYEG machinery. In the current model, periplasmic chaperones such as SurA bind the nascent OMPs as they emerge from the Sec translocon and shuttle them across the periplasm to prevent their aggregation [3], [4], [5]. A multiprotein complex in the outer membrane called β-Barrel Assembly Machine (BAM) then mediates OMP folding and insertion [6].

In *E. coli*, the BAM complex is composed of BamA, a β-barrel OMP itself previously known as Omp85/YaeT [6], [7], [8], and the lipoproteins BamB, C, D and E (previously YfgL, NlpB, YfiO and SmpA respectively) [6], [9]. BamA appears to be the central component of the complex and is conserved in diderm bacteria as well as mitochondria and chloroplasts where it also plays a role in insertion of β-barrel proteins in the outer membrane of these organelles [10]. Deletion of the *bamA* genes is lethal, and deletion of *bamD* is also lethal in *E. coli* and *Neisseria* but tolerated in *Salmonella* [6], [7], [11], [12], [13]. Conversely, deletion of *bamB, bamC* or *bamE* results in outer membrane permeability defects with *bamB* deletion producing the strongest phenotype of the three [6], [9].

The molecular mechanisms that mediate OMP folding and insertion by BAM are only beginning to emerge. In addition to its β-barrel domain, BamA contains a large periplasmic domain with five POlypeptide TRanslocation Associated (POTRA) repeats. Crystallographic and solution scattering studies of the periplasmic domain of BamA revealed a structure with two rigid arms formed by POTRA1-2 and POTRA3-5 joined by a linker that affords a great deal of flexibility to the structure [14], [15], [16]. Analysis of the crystal contacts suggested that substrate OMPs may bind the edges of POTRA β-sheets nucleating β-strand formation in the nascent OMPs [14], [15]. NMR studies with POTRA domains and peptides derived from OMP β-barrels were consistent with this hypothesis [17].

**Table 1:** Data Collection, Phasing and Refinement Statistics.

	Native BamB	SeMet BamB
**Data Collection**		
Space Group	P3_2_21	P3_2_21
Unit Cell: *a* = *b* (Å) *c* (Å)	82.98 83.38	82.87 81.65
Wavelength (Å)	1.0000	0.9792
Resolution (Å)^a^	50.0–1.85 (1.92–1.85)	50.0–2.4 (2.49–2.40)
*R*_sym_ b (%)	3.6 (9.0)	7.3 (20.5)
*I*/s	30.2 (16.5)	23.9 (10.4)
Data Completeness (%)	99.4 (99.5)	100 (100)
Redundancy	3.5 (3.6)	5.7 (5.7)
**Phasing**		
FOM before DM^c^		0.38 (0.28)
FOM after DM		0.68 (0.41)
**Refinement Statistics**		
Resolution (Å)	33.0–1.85 (1.91–1.85)	
Number of reflections	28674	
Protein atoms (no H)	2643	
Water molecules	402	
*R*_work_ (%)	16.0 (18.6)	
*R*_free_ d (%)	20.5 (25.8)	
RMS dev. bonds (Å)	0.011	
RMS dev. angles (°)	1.56	

a Values in parentheses are for the highest-resolution shell.

b R_sym_ = ∑_h_∑_i_ |(I_i_(h)-<I(h)>|/∑_h_∑_I_ I_i_(h), where I_i_(h) is the I-th measurement of reflection h, and <I(h)> is the weighted mean of all measurements of h.

c Figure Of Merit before Density Modification.

d R_work_  =  ∑|F_obs_-F_calc_|/∑F_obs_ where F_obs_  =  observed structure factor amplitude and F_calc_  =  structure factor calculated from model. R_free_ is computed in the same as R_work_, but using the test set of reflections.

**Structures of isolated BAM subunits have been determined** [14], [15], [16], [18], [19], [20], [21], [22], [23], [24], and the structure of a BamCD complex has also been reported [25]. However, despite this wealth of structural information, little is known about the function of the lipoprotein components of BAM. Here we show that expression of *Pseudomonas aeruginosa bamB* can complement the membrane phenotype of Δ*bamB E. coli* (also reported in [26]) suggesting that the functionally relevant features are conserved in these two proteins despite the relatively low (30%) sequence identity. We report the crystal structure of *Pseudomonas aeruginosa* BamB refined to 1.85Å resolution. Comparison to the existing *E. coli* structures reveals the conserved structural features and their importance for BamB function are systematically tested by mutagenesis.

**Figure 1:**
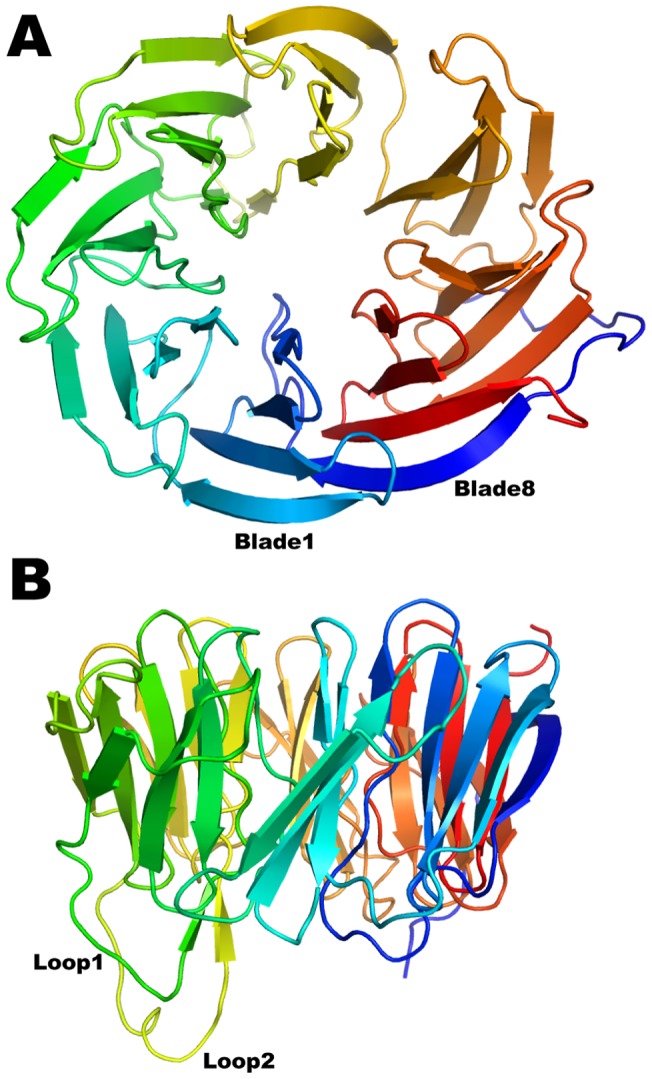
Structure of BamB. Top (A) and side (B) view of the eight bladed β-propeller fold of BamB color-ramped from blue (N-terminus) to red (C-terminus). The first and eighth blades are labeled for reference. Two protruding loops connecting blades 3 to 4 and 4 to 5 are labeled Loop 1 and 2 and colored green and yellow respectively.

## Materials and Methods

### Cloning, Expression and Purification of paBamB

The gene fragment for mature paBamB (lacking the 18 amino acid signal sequence) was amplified by polymerase chain reaction (PCR) from genomic *P. aeruginosa* PA01 DNA using AccuPrime Pfx DNA polymerase (Invitrogen). The PCR primers incorporated *NcoI* and *XmaI* restriction sites at the N- and C-terminus, respectively, and mutated the N-terminal cysteine residue to alanine. The digested and purified gene fragment was ligated into an engineered pET28 vector that incorporates an N-terminal 6xHis-tag and the Tobacco etch virus (TEV) protease cleavage site to the cloned gene. The resulting plasmid, pMS327, was sequenced to verify the correctness of the construct.

**Figure 2:**
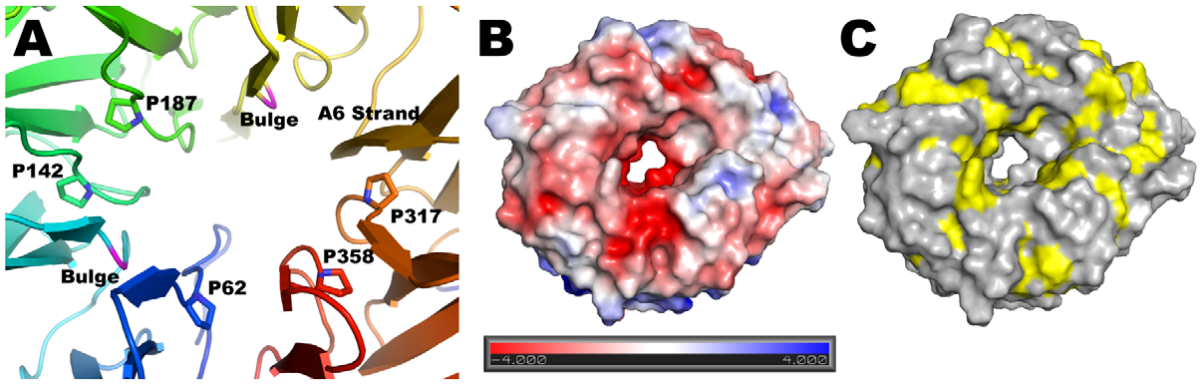
Central pore and surface characteristics of BamB. (A) Seven of the A strands lining the central pore are irregular due to conserved proline residues and two bulges (shown in magenta). The A6 strand has a regular b conformation along the entire pore (shown in orange). (B and C) Top view of a surface representation of BamB colored by electrostatic surface potential (B) or hydrophobicity (C) showing the central pore in the structure.

*Escherichia coli* BL21(DE3) cells (Novagen) transformed with pMS327 were selected on kanamycin LB plates. An overnight culture from a single colony was used to inoculate 6× 1L LB medium supplemented with 50 µg/mL kanamycin. The cultures were then grown at 37°C to OD_600_ of 0.6, at which point the cells were induced with 0.4 mM isopropyl-β-D-thiogalactopyranoside (IPTG, Gold Bio Technology Inc.). The protein was expressed for 2 hrs at 37°C. Pelleted cells were re-suspended in lysis buffer (25 mM Tris pH 8.0) and Complete EDTA-free protease inhibitor cocktail (Roche). Cell lysis was performed by sonication, and the suspension was supplemented with NaCl to final concentration of 0.3 M. After the centrifugation (16,000 rpm for 30 min at 4°C), the soluble fraction was purified using a Ni-NTA column (Qiagen) pre-equilibrated with buffer A (25 mM Tris pH8, 150 mM NaCl). Protein bound to the column was washed with 2 column volumes of buffer A, followed by a wash with 20 column volumes of buffer B (25 mM Tris pH 8, 150 mM NaCl, 25 mM Imidazole). The protein was eluted with buffer C (25 mM Tris pH 8, 150 mM NaCl, 200 mM Imidazole). Eluted protein fractions were incubated with His-tagged-TEV protease for 24 h at 4°C to cleave His tag followed by overnight dialysis at 4°C in buffer A. The sample was then treated with Ni-NTA beads to remove the tag, His-tagged-TEV, and any remaining un-cleaved His-BamB. Finally the proteins was subjected to size exclusion chromatography (HiLoad 26/60 Superdex 200, Amersham Pharmacia Biotech) in buffer A. BamB eluted in a single peak and was concentrated to 10 mg/mL for crystallization and stored at −70°C. Seleno-methionine labeling of BamB was accomplished as described previously [15] and purified with the same protocol.

**Figure 3:**
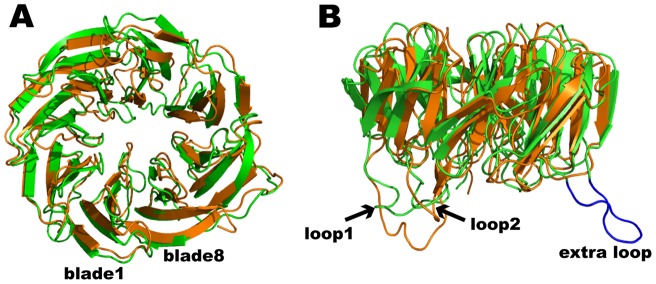
Comparison of BamB from *Pseudomonas aeruginosa* and *E. coli*. (A) Top view of paBamB (orange) superimposed on ecBamB (green, PDB ID: 3P1L). (B) Side view of the same superposition. Loops 1 and 2 are conserved in both proteins whereas ecBamB displays and additional loop connecting blades 2 and 3 (extra loop, shown in blue).

### Protein Crystallization and Data Collection

BamB was crystallized by the hanging drop vapor diffusion method (1.5 µl protein: 1.5 µl precipitant) at 16°C in the PEG/Ion HT screen (Hampton Research). Conditions refined to 10% Tacsimate pH 7.0 and 10% PEG 3,500 yielded single plates. Crystals were harvested, cryo-protected in the solution containing mother liquor and 30% PEG, and were flash frozen in a stream of N_2_ at 100°K. Crystals of seleno-methionine substituted BamB were obtained in similar conditions and cryo-protected in mother liquor.

**Figure 4:**
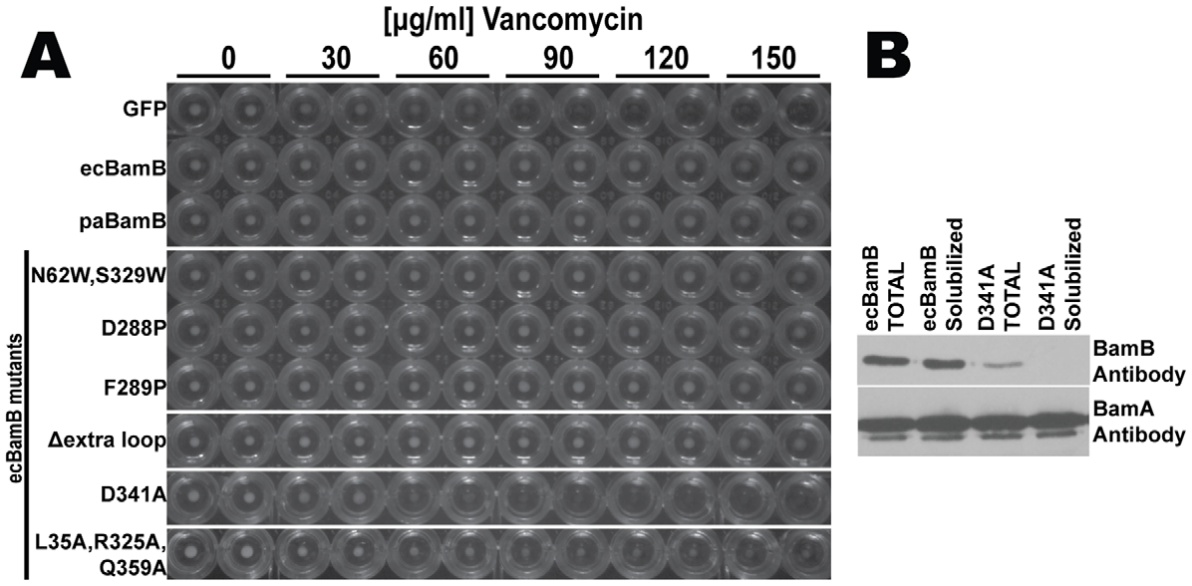
Complementation of Δ*bamB* phenotype in *E. coli*. (A) Cells expressing control plasmid (GFP) do not grow at vancomycin concentrations higher than 60 µg/ml (no cell pellets in the round bottom wells) whereas plasmids expressing wild type *E. coli* or *Pseudomonas* BamB (ecBamb and paBamB respectively) complement the Δ*bamB* phenotype and grow at higher vancomycin concentrations (at least 150 µg/ml). All the ecBamB mutants tested were able to complement the Δ*bamB* phenotype except D341A. (B) Western blots of total and detergent solubilized *E. coli* Δ*bamB* cells expressing wild type (ecBamB) or mutant (D341A) BamB from a plasmid. Equivalent cell loads (controlled by probing with BamA antibody) show that the ecBamB D341A mutant expresses at a lower level and fails to properly assemble in the membrane (see text for details).

Data collection on native and seleno-methionine substituted BamB was performed at BeamLine 8.2.1 of the Advanced Light Source in Lawrence Berkeley National Laboratory. Data were indexed and integrated with HKL2000 [27]. X-ray data collection statistics for both data sets are shown in Table 1.

**Figure 5:**
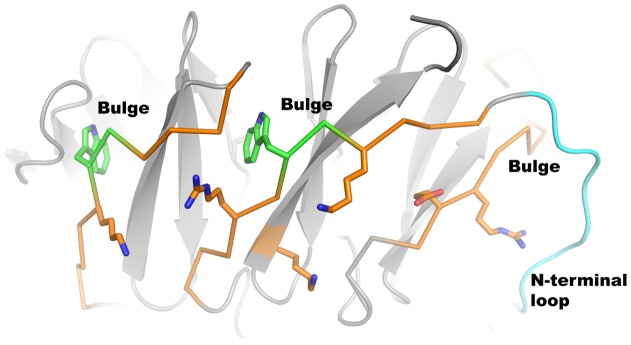
Features preventing β-augmentation in D strands of BamB. Three D-strands of BamB are shown as an orange alpha-carbon trace displaying three features that disfavors β-augmentation: The tryptophan in the docking motifs introduces a bulge in the strand (green) and strategically placed charged residues would have to be buried for extension of the sheets. The N-terminal loop also protects one D strand from b-augmentation.

### Structure Determination

A single wavelength dataset to 2.4 Å resolution collected from seleno-methionine substituted BamB was used to solve the structure using the AutoSol module of Phenix [28]. Three sites were readily identified (*Pseudomonas* BamB has three methionines) and used to calculate SAD phases followed by density modification. This procedure resolved the ambiguity of the enantiomeric space group and yielded a readily interpretable electron density map. A partial model generated with the AutoBuild module of Phenix was manually rebuilt and completed using Coot [29]. Iterations of refinement in Phenix and manual rebuilding were carried out until no further improvement of the R-factor could be achieved. Final statistics are shown in Table 1. Atomic coordinates and structure factors have been deposited in the Protein Data bank under PDB ID: 4HDJ.

**Figure 6:**
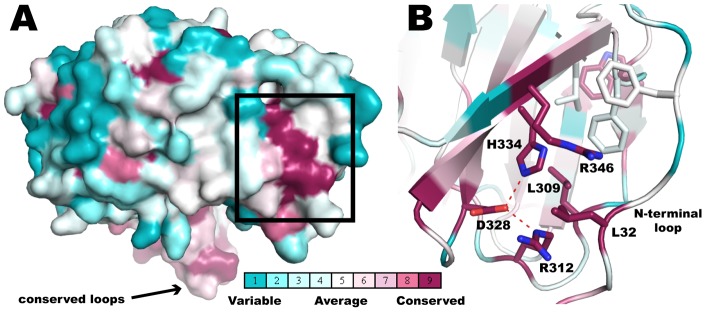
BamB Surface Residue Conservation Analysis. 335 BamB sequences were subjected to Consurf analysis to create conservations scores (see text for details). The conservation is color coded as follows: conservation scores are segregated into 9 discrete bins, such that bin 1 contains the most rapidly evolving residues (most variable) and is colored blue; bin 5 contains residues with average rate of evolution (colored white); and bin 9 has the most conserved residues and is colored dark purple. (A) Side view of a surface representation of BamB displaying a patch of conserved residues on the side of the propeller. (D) Close up of the region framed in (C) detailing the interactions at the groove between blades 6 and 7 and the N-terminal loop.

### Sequence Conservation Analysis

Starting with the *P. aeruginosa* sequence, we generated a hidden Markov model for BamB running five iterations of HMMER [30] against the TrEMBL database with a conservative E value cutoff of 1e^−80^ for inclusion into the model. We chose this conservative cutoff to avoid “contaminating” the model with sequences from PQQ-dependent ADHs that contain the tryptophan docking motif, and also to avoid including BamB sequences from very distantly related organisms, where the interfaces for binding partners may have co-diverged. The resulting alignment was edited manually to eliminate sequences that did not contain the invariant cysteine after the signal sequence. We also eliminated redundancy by eliminating sequences identical to at least one other sequence in the alignment. The resulting 335 sequences were subjected to conservation analysis. The Consurf server [31] used to carry out the analysis uses Bayesian methods to calculate amino acid conservation scores. The scores are then normalized such that the average score is zero and the standard deviation is one. Therefore, rapidly evolving or “variable” residues will have a negative score whereas slowly evolving or “conserved” residues have positive values. To visualize the relative conservation of surface exposed residues the scores were mapped on the structure of BamB and colored using the standard Consurf scheme where non-conserved (rapidly evolving) residues are cyan; residues with average rate of evolution are white and the most conserved residues are dark purple.

### Construction of the *E. coli* Δ*bamB* strain

The null *bamB* mutant strain MCS4 was constructed by chromosomal disruption of the *bamB* gene according to Datsenko and Wanner [32]. Briefly, PCR product of the template pKD4 plasmid was generated using the following primers (U619: 5′-CGAAATGATGCAGATGAAAATTAATAATTTGTCCATCTGAGAGGGACCCG TGTGTAGGCTGGAGCTGCTTC-3′ and L619: 5′-GAAAACGGCCCCTGTCCAGGAGCCGTTTTCAAAGTGAACGACAGAGACGACATATGAATATCCTCCTTAGT-3′). The PCR product was then gel purified and used to transform *E. coli* strain BW25113 expressing λ Red recombinase from the plasmid pKD46. The transformants were selected on kanamycin LB plates. The clones without *bamB* gene were verified using PCR and the *bamB* deletion cassette was then moved into MC4100 using P1 phage transduction. The kanamycin resistance cassette from this strain was removed using plasmid pCP20 to produce the *ΔbamB E. coli* strain used in this study (MCS4). Whereas the original MC4100 strain carries a mutation that makes it sensitive to arabinose, natural revertants are easily isolated. Our *ΔbamB E. coli* is not sensitive to arabinose and can thus be used in complementation assays with arabinose inducible plasmids.

### Complementation Plasmid Construction

The gene for wild type *E. coli* BamB was amplified and ligated into pZS21 [33] resulting in plasmid pMS734. The promoter was then replaced with araBAD to generate a low copy number plasmid with an inducible promoter. Briefly, the araBAD promoter was PCR amplified from pBAD (Invitrogen) using the primers U781: 5′-TCGAGAAACCAATTGTCCATATTG-3′ and L781: 5′-GGTACCTTCCTCCTGTTAGCCCAAAA-3′). The PCR product was XhoI/KpnI digested and ligated into pMS734 digested with the same enzymes resulting in plasmid pMS829. This plasmid was then used to produce all the ecBamB mutants described in the manuscript using the Quick Change Mutagenesis procedure (Invitrogen) and appropriate sets of primers (see supplement for more detail). The mature *Pseudomonas aeruginosa bamB* gene (without signal sequence, paBamB) was obtained by PCR amplification from genomic DNA with primers U668:5′-TTAAGCGGCTGTAGCAGCAACAGCAAGAAGGAACTCC-3 and L668-1:5′-GATGCCTCTAGATTAGTGATGATGATGATGATGCCCGGGGCG-3′. This fragment was seamlessly ligated into pMS829 after the signal sequence of *E. coli bamB* by In-Fusion cloning (Clontech) resulting in plasmid pMS907. All *bamB* containing constructs have a C-terminal His-tag without a TEV cleavage site. A negative control plasmid (pMS906) was constructed by replacing ecBamB with Green Fluorescent Protein (GFP) amplified from plasmid pZS21-GFP obtained from Dr. Silhavy (Princeton University).

### Complementation Assay/Minimal Inhibitory Concentration

Strain MCS4 (*ΔbamB E. coli*) was transformed with complementing plasmids and selected on kanamycin LB plates at 37°C overnight. A colony from each plate was grown in 5 ml LB culture in the presence of kanamycin and 0.1% arabinose (to induce expression of the plasmid encoded gene) until mid-log phase. The cell suspension was diluted to OD_(600)_ = 0.1 and mixed with an equal volume of LB containing varying amounts of vancomycin in a round bottom 96 well plate. The plates are then incubated at 37°C overnight with agitation, and spun down to evaluate cell growth (the size of cell pellets).

## Results and Discussion

### Structure Determination and paBamB Model

BamB is a membrane-associated lipoprotein with lipids attached to the N-terminal cysteine in the mature protein predicted to anchor it to the membrane [6], [34]. A lipid-free, soluble form of mature *Pseudomonas aeruginosa* BamB (paBamA, amino acids 19–380) was expressed in *E. coli*, purified to homogeneity and crystallized as described in Methods. The crystals belong to trigonal space group P3_2_21 and contain a single paBamB molecule per asymmetric unit. Similar conditions also yielded crystals of seleno-methionine substituted BamB. A single wavelength dataset at the selenium absorption peak was collected from these crystals and used to determine the structure by SAD techniques as described in Methods. The model was then refined against a native dataset to 1.85Å resolution. The final model contains amino acids 28 to 380 (plus two extra amino acids, PG, at the C-terminus resulting from the cloning procedure) with excellent refinement statistics as shown in Table 1.

BamB adopts an eight-bladed β-propeller fold (Figure 1). The blades, sometimes referred to as W-motifs, are arranged radially around a pseudo eight-fold symmetry axis. Four antiparallel β-strands named A–D constitute each blade with the A strand being closest to the pseudo-symmetry axis (A1 thus refers to the A strand of Blade1). The eighth blade is formed by three strands from the C-terminus (A–C, red in Figure 1) and the D-strand from the N-terminus (blue in Figure 1) mediating closure of the ring. The connections between the propeller strands are generally short with the exception of those connecting D3 to A4 (loop 1, green in Figure 1B), and D4 to A5 (loop 2, yellow in Figure 1B). These two loops protrude from the bottom of the propeller and interact with one another through two short parallel β-strands outside of the propeller.

BamB appears related to PQQ-dependent dehydrogenases (PQQ-DHs), sharing features such as the eight bladed β-propeller fold and the presence of repeating “tryptophan-docking motifs” with a consensus sequence AX[D/N]XXT**G**[D/E/K]XX**W**, where X can be any amino acid and the G and W are invariant (Figure S1A; described in detail in [35], [36]). Structurally, the tryptophan residues occur at the beginning of the D strand of a blade and its side chain “docks” against the planar peptide bond of the invariant glycine in the motif of the next blade forming a stabilizing girdle (Figure S1B). BamB is the first structure aside from PQQ-DHs to display these inter-blade tryptophan-docking interactions.

With the exception of A6, the A strands are irregular, making only 2 to 3 regular β-type hydrogen bonds with the B strands. This is rather unusual but is also observed in PQQ dependent dehydrogenases as well as *E. coli* BamB (see below). In paBamB, the irregularities are brought about by proline residues in A1, A3, A4, A7 and A8, and β-bulges in A2 and A5 that occur in the middle of the strands forming a ring around the pseudo eight-fold symmetry axis (Figure 2A). Despite these irregularities in the inner strands, the propeller displays a central pore that is about 20Å wide at the “top” and narrows to about 8Å at the center. The pore has a negative electrostatic surface potential (Figure 2B) and is filled with ordered water molecules in the crystal structure. The only regular β-strand in the pore is A6, which makes 6 β-type contacts with B6 and offers 3 carbonyls and 2 amide NH to the pore in a regular β-strand conformation. The main constraint to the diameter of this pore is provided by the side chains of non-conserved residues E61 and R357, the latter hydrogen bonding the carbonyl of V276 in the middle of strand A6 (Figure S2). The surface exposed residues of BamB are generally hydrophilic. However, the top of the propeller displays grooves lined with hydrophobic residues (Figure 2C) that alternate with grooves rich in charged residues (Figure 2B). Surfaces involved in protein-protein interactions are more hydrophobic than exposed surfaces [37]. Therefore, the grooves lined with hydrophobic residues on the top surface of BamB may represent sites of interaction with other BAM components, assembly chaperones or nascent OMPs.

### Comparison of *E. coli* and *P. aeruginosa* BamB Structures and Functional Evaluation of their Structural Features

The structures of paBamB and ecBamB (*E. coli* BamB) superimpose with an RMS deviation of 1.8 Å for 286 matching Cα atoms (Figure 3A) highlighting a high degree of structural conservation despite a relatively modest 30% sequence identity. All seven Trp-docking motifs are conserved. The Loops 1 and 2 (connecting D3-A4 and D4-A5) protruding from the bottom of the protein are present in both homologs, although they do not adopt regular β-strand conformations in the crystal structures of ecBamB (Figure 3B). Working with *E. coli* BamB, Misra and coworkers reported that mutation of conserved residues L173, L175 and R176 in loop L1 (equivalent to L179, L181 and R182 in paBamB) impaired binding to BamA resulting in a deficient BAM phenotype [26]. The double mutants equivalent to L179/R182 and L181/R182 were also deficient, whereas single mutations were not enough to display a phenotype. They also reported that mutation to alanine of D227A and D229A in loop L2 (equivalent to D233 and D235 in paBamB) where deficient at co-precipitating BamA. Moreover, mutation to cysteine of the non-conserved residues S172 and S226 in loops L1 and L2, respectively, allowed crosslinking of BamB to BamA using the bifunctional crosslinker N-succinimidyl 3-(2-pyridyldithio) propionate (SPDP). Despite their distance in the sequence, these residues cluster together in the structure, indicating that the protruding loops 1 and 2 contain conserved residues that mediate the BamB-BamA interaction.

Residues 99–108 in ecBamB form an extended loop connecting D1–A2 that is not present in paBamB (blue in Figure 3B). To test the functional importance of this and other structural features in BamB, we created an *E. coli bamB* null mutant (Δ*bamB*) using standard recombineering techniques [32]. As reported previously, Δ*bamB* mutants are viable but display membrane permeability defects that render them more sensitive to antibiotics such as rifampcin and vancomycin [38]. This phenotype can be complemented with a plasmid borne copy of wild type *bamB*. Expression of an ecBamB mutant where the loop residues 99–108 were replaced by a single glycine also complemented Δ*bamB* (Figure 4). Furthermore, expression of paBamB (which does not contain this extra loop) is also able to complement the *E. coli* Δ*bamB* strain (Figure 4) [26]. These experiments indicate that the 99–108 loop is not required for BamB function and further indicates that only features conserved in both *E. coli* and *Pseudomonas* BamB are necessary for function.

The A6 strand is the only regular β-strand lining the pore of paBamB. All the other inner (A) strands contain prolines or β-bulges that interrupt the regular β-hydrogen bonding pattern (Figure 2A). This unusual structural feature is conserved in ecBamB (in ecBamB there is a bulge in A2 and prolines in the other irregular strands). We reasoned that A6 may be the only regular β-strand in the center of BamB to specifically interact by β-augmentation [39] with either substrate OMPs or other components of BAM. However, two point mutants of ecBamB, D288P and F289P, designed to disrupt the A6 regular β-strand complemented the Δ*bamB* strain (Figure 4) indicating that a single regular strand is not required for a normal BamB phenotype.

The central pore of ecBamB is similar in size and shape to that of paBamB. In contrast, the structurally related PQQ-DHs do not display such a pore. In these enzymes, all the A strands are irregular due to their side chains projecting towards the central pseudo-eight fold axis and packing closely to fill the space [35]. In addition, the N-terminus of PQQ-DHs folds into a short helix and a series of loops that sit on the central opening of the propeller blocking it completely. We thus hypothesized that the central pore in BamB may have a functional role. The size of the pore appears to be large enough to accommodate an extended polypeptide, opening the possibility that substrate nascent OMPs could be threaded through this opening. We thus created an ecBamB N62W/S329W double mutant designed to replace small side chains in the pore with bulky residues that would obstruct the opening (Figure S3). However, this mutant was able to fully complement the Δ*bamB* strain (Figure 4). Whereas it is possible that the double Trp mutant failed to fully occlude the central pore in ecBamB, the mutations should at least severely narrow the lumen. Therefore the absence of a phenotype associated with the double Trp mutants suggest that an open pore in the center of BamB is not required for function.

The BAM complex is thought to interact with nascent OMP in an unfolded state [8]. Crystallographic studies of the POTRA domains of BamA suggested that binding of substrate OMPs might occur at the edge of β-sheets, nucleating formation of β-strands in the nascent OMPs by β-augmentation [14], [15]. NMR studies of POTRA domains and OMP-derived peptides were consistent with this hypothesis [17]. When it was recognized that BamB could have a β-propeller fold similar to PQQ-binding enzymes it was suggested that the outer strands of the propeller could also bind nascent OMPs by β-augmentation or interact with β-strands in BamA [20], [40]. However, most β-rich proteins have evolved features to avoid extension of their β-sheets because promiscuous edge-to-edge interactions would lead to aggregation as observed in amyloid fibers [41]. The features most frequently seen in the outer strands of β-propellers are (i) a charged amino acid in the middle of the strand that would be buried by the sheet extension; and (ii) the presence of β-bulges that disrupt regular β-sheet interactions by introducing a local twist to the strand [41]. All eight D strands in paBamB contain at least one charged residue in the middle (and as many as three) and seven of the D strands also contain a β-bulge provided by the Trp-docking motif (Figure 5) suggesting that outward extension of the BamB propeller β-sheets is unlikely. Furthermore, we were not able to detect any binding of ecBamB to peptides derived from the β-strands of LamB or BtuB in Isothermal Titration Calorimetry (ITC) experiments (Figure S4). Therefore, whereas some crystals of ecBamB are stabilized by β-augmentation lattice contacts [20], we conclude that under physiological protein concentrations, nascent OMP binding to BamB by β-augmentation is unlikely.

Efficient folding and insertion of OMPs *in vivo* appears to depend on a consensus sequence at the C-terminus of the β-barrel [42], [43]. The last residue in the β-barrel is almost always F or W and, starting from the C-terminus, positions 3, 5, 7 and 9 are hydrophobic residues with Y frequently occupying position 3 [43]. Thus, this “OMP tag” appears to be important for one or more mechanistic steps in OMP folding and insertion. It has been proposed, based on analysis if the lattice contacts stabilizing the crystal of ecBamB, that a surface cavity in BamB could mediate recognition of the aromatic C-terminal “OMP tag” [20]. This surface cavity is not conserved in paBamB primarily because M193 replaces A206 in ecBamB and the larger side chain occludes the cavity. Since paBamB can complement the Δ*bamB* phenotype, only conserved features are likely to be important for function. Furthermore, ITC experiments failed to show any binding of ecBamB to a peptide of sequence EYTLSGSYTF corresponding to the C-terminus of the OMP BtuB (Figure S4) suggesting that BamB is not a receptor for the “OMP tag”.

Sequence analysis of 335 non-redundant homologous sequences of BamB (see details in Experimental Procedures) reveals only two patches of surface-exposed conserved residues. One cluster is in the loops 1 and 2 that, as discussed above, mediate interaction with BamA [26]. The second patch of conserved surface residues occurs on the side of the propeller between blades 6 and 7 (Figure 6A and B). In paBamB, H334 and R312 are within hydrogen bonding distance of D328 creating a strictly conserved triad that packs against the side chains of R346 and L309. The N-terminus of paBamB forms an extended loop that folds over the side of the propeller and contributes the conserved L32 to the site (Figure 1 and 6B). This creates a groove where the conserved R346, L32 and R312 are exposed to the surface. This feature is conserved in ecBamB, with the only difference being that R346 is replaced with a glutamine in ecBamB but serving a similar hydrogen-bonding role. We thus hypothesized that this may represent a binding interface in BamB. Consistent with this idea, it has been shown that the β-propeller domain of clathrin binds different cargo proteins in a groove between propeller blades [44]. A similar mode of binding was observed in the complex between Matrix Metalloproteinase 2 and its inhibitor TIMP2 [45]. These “peptide-in-groove” interactions are thought to represent a general mechanism for propeller binding to target proteins [44].

To test this hypothesis, we created a triple mutant of ecBamB where L35, R325 and Q359 were mutated to alanine (corresponding to L32, R312 and R346, respectively, in paBamB). However, the triple mutant was able to complement the Δ*bamB* phenotype, albeit with a somewhat reduced growth rate at the higher antibiotic concentrations (Figure 4A). Western blot analysis of cells complemented with the triple mutant showed significantly reduced levels of BamB in the membranes (Figure 4B), which explain the reduced growth observed in the complementation assay. We also tested the effect of mutating the conserved triad. In this case, ecBamB D341A (corresponding to the paBamB D328) was unable to complement the *E. coli* Δ*bamB* phenotype. However, western blots showed that this mutant failed to reach the membrane and instead accumulated as an insoluble product (data not shown). Whereas we cannot rule out a role of the conserved surface residues in BamB protein-protein interactions, the results suggest that these conserved residues, in particular the HDR triad, are crucial for proper folding or biogenesis of BamB.

Taken together, the structural analysis and mutagenic testing of BamB suggests that its main function is to bind and modulate BamA, rather than directly interact with nascent OMPs. This notion is supported by the modular character of the BAM complex, where BamB appears to interact only with BamA whereas BamC, D and E form a separable module [5], [46], [47]. The periplasmic domain of BamA contains five POTRA motifs displaying a great deal of flexibility around a hinge point located between POTRA2 and 3 that may play a role in OMP folding or insertion [14], [15], [16]. BamA mutants where individual POTRA motifs were deleted one at a time showed that deletion of POTRA2, 3 or 4 results in loss of BamB binding [14]. Therefore, we speculate that BamB interacts directly with POTRA 2 and 3 of BamA and modulates the flexibility of its periplasmic domain increasing its efficiency.

## Supporting Information

Figure S1 **The Tryptophan Docking Motif (A) Close up of a Trp docking motif in BamB.** The amide NH of A80 from the motif in one blade (green) hydrogen bonds with the carbonyl of W90 in the same motif. The side chain of W90 docks against the planar peptide bond between T125 and G126 in the motif on the next blade (orange). The indole NH of W90 also hydrogen bonds with the main chain carbonyl from E123 in the next blade. The tryptophan and the glycine are invariant residues and the interactions of the tryptophan with the main chain of both blades are the hallmark of the motif. For clarity, several side chains are not displayed. (B) Distribution of Trp docking motifs on BamB. The motifs stabilize seven of the eight blades in the BamB propeller forming a Trp girdle.
(TIF)

Figure S2 **The Central Pore in BamB.** A semitransparent surface representation of BamB, viewed down the central pore, reveals the packing of side chains lining the pore. E61 and R357 provide the main constrain to the volume of the pore. However, different rotamers could enlarge its capacity. In the crystal structure, the side chain of R357 hydrogen bonds the carbonyl of V276 in the A6 strand.
(TIF)

Figure S3 **Mutations Blocking the Central Pore in BamB.** Modeling of side chains in ecBamB N62W/S329W double mutant result in occlusion of the central pore in the protein.
(TIF)

Figure S4 **Representative ITC traces for the titration of β-barrel derived peptides on BamB.** For each panel (A–D), the top plot represents the heat evolved after each injection (µCal/sec) vs time; while the bottom section shows the enthalpy (kcal/mol) as a function of peptide:ecBamB molar ratio (note: “the peptide into buffer titrations (panels B and D) show arbitrary numbers for “molar ratio” as no protein is present). The titration of 203 µM LamB peptide (sequence DFHGYARSGIGWT) on 25 µM ecBamB (A) is essentially the same as the titration of the same peptide solution into buffer (B). Similarly, titration of 1.5 mM BtuB peptide (sequence EYTLSGSYTF) on 17 µM ecBamB (C) was indistinguishable from the titration of the same peptide solution into buffer (D). We thus conclude that the observed signals are due to the heat of peptide dilution and no additional heat due to peptide binding to ecBamB is observed under these conditions. Titration of BtuB peptide was done at a large excess of peptide to account for the possibility of multiple binding sites. However, experiments carried out at peptide:BamB molar ratios in the range 1 to 3.5 were identical (data not shown), also indicating no detectable binding of the peptide to ecBamB. ***Isothermal Titration Calorimetry method details:*** Purified ecBamB was extensively dialyzed against buffer A (25 mM TrisHCl pH 8.0, 150 mM NaCl) prior to the ICT experiments. The peptide derived from the β-barrel of LamB: DFHGYARSGIGWT (>98% pure, synthesized by Creative Peptides), was dissolved in 50% DMSO and then subjected to buffer exchange on a PD-G10 column equilibrated in buffer A. Final peptide concentration was measured by UV absorbance at 280 nm using an extinction coefficient of 6,990 M^−1^ cm^−1^ (calculated from the amino acid sequence). The peptide derived from the C-terminal strand of BtuB: EYTLSGSYTF (>98% pure, synthesized by Anaspec) was soluble in buffer A but was also passed through a PVDF 0.1 µm filter equilibrated in buffer A. Final peptide concentration was measured by UV absorbance at 280 nm using an extinction coefficient of 2,980 M^−1^ cm^−1^ (calculated from the amino acid sequence). All ITC measurements were carried out on an iTC 200 instrument (GE) at 25°C. The sample cell (203 µL) was filled either with buffer A to obtain the heat of peptide dilution (negative control) or with ecBamB to monitor the interaction. The titration of peptides was performed with 21 2 µL injections (except the 1^st^ and the last, injecting 0.2 µL and 1.6 µL titrant, respectively).
(TIF)

Table S1 **Primers used in the construction of plasmids.**
(DOC)

## References

[1] Nikaido H (2003) Molecular basis of bacterial outer membrane permeability revisited. Microbiol Mol Biol Rev 67: 593–656. 14665678 10.1128/MMBR.67.4.593-656.2003 PMC309051

[2] Schulz GE (2003) Transmembrane beta-barrel proteins. Adv Protein Chem 63: 47–70. 12629966 10.1016/s0065-3233(03)63003-2

[3] Ruiz N, Kahne D, Silhavy TJ (2006) Advances in understanding bacterial outer-membrane biogenesis. Nat Rev Microbiol 4: 57–66. 16357861 10.1038/nrmicro1322

[4] Sklar JG, Wu T, Kahne D, Silhavy TJ (2007) Defining the roles of the periplasmic chaperones SurA, Skp, and DegP in Escherichia coli. Genes Dev 21: 2473–2484. 17908933 10.1101/gad.1581007 PMC1993877

[5] Hagan CL, Kim S, Kahne D (2010) Reconstitution of outer membrane protein assembly from purified components. Science 328: 890–892. 20378773 10.1126/science.1188919 PMC2873164

[6] Wu T, Malinverni J, Ruiz N, Kim S, Silhavy TJ, et al (2005) Identification of a multicomponent complex required for outer membrane biogenesis in Escherichia coli. Cell 121: 235–245. 15851030 10.1016/j.cell.2005.02.015

[7] Voulhoux R, Bos MP, Geurtsen J, Mols M, Tommassen J (2003) Role of a highly conserved bacterial protein in outer membrane protein assembly. Science 299: 262–265. 12522254 10.1126/science.1078973

[8] Voulhoux R, Tommassen J (2004) Omp85, an evolutionarily conserved bacterial protein involved in outer-membrane-protein assembly. Res Microbiol 155: 129–135. 15143770 10.1016/j.resmic.2003.11.007

[9] Sklar JG, Wu T, Gronenberg LS, Malinverni JC, Kahne D, et al (2007) Lipoprotein SmpA is a component of the YaeT complex that assembles outer membrane proteins in Escherichia coli. Proc Natl Acad Sci U S A 104: 6400–6405. 17404237 10.1073/pnas.0701579104 PMC1851043

[10] Gentle IE, Burri L, Lithgow T (2005) Molecular architecture and function of the Omp85 family of proteins. Mol Microbiol 58: 1216–1225. 16313611 10.1111/j.1365-2958.2005.04906.x

[11] Onufryk C, Crouch ML, Fang FC, Gross CA (2005) Characterization of six lipoproteins in the sigmaE regulon. J Bacteriol 187: 4552–4561. 15968066 10.1128/JB.187.13.4552-4561.2005 PMC1151791

[12] Malinverni JC, Werner J, Kim S, Sklar JG, Kahne D, et al (2006) YfiO stabilizes the YaeT complex and is essential for outer membrane protein assembly in Escherichia coli. Mol Microbiol 61: 151–164. 16824102 10.1111/j.1365-2958.2006.05211.x

[13] Fardini Y, Trotereau J, Bottreau E, Souchard C, Velge P, et al (2009) Investigation of the role of the BAM complex and SurA chaperone in outer-membrane protein biogenesis and type III secretion system expression in Salmonella. Microbiology 155: 1613–1622. 19372159 10.1099/mic.0.025155-0

[14] Kim S, Malinverni JC, Sliz P, Silhavy TJ, Harrison SC, et al (2007) Structure and function of an essential component of the outer membrane protein assembly machine. Science 317: 961–964. 17702946 10.1126/science.1143993

[15] Gatzeva-Topalova PZ, Walton TA, Sousa MC (2008) Crystal structure of YaeT: conformational flexibility and substrate recognition. Structure 16: 1873–1881. 19081063 10.1016/j.str.2008.09.014 PMC2642521

[16] Gatzeva-Topalova PZ, Warner LR, Pardi A, Sousa MC (2010) Structure and flexibility of the complete periplasmic domain of BamA: the protein insertion machine of the outer membrane. Structure 18: 1492–1501. 21070948 10.1016/j.str.2010.08.012 PMC2991101

[17] Knowles TJ, Jeeves M, Bobat S, Dancea F, McClelland D, et al (2008) Fold and function of polypeptide transport-associated domains responsible for delivering unfolded proteins to membranes. Mol Microbiol 68: 1216–1227. 18430136 10.1111/j.1365-2958.2008.06225.x

[18] Albrecht R, Zeth K (2011) Structural basis of outer membrane protein biogenesis in bacteria. J Biol Chem. 10.1074/jbc.M111.238931 PMC3149369 21586578

[19] Endo T, Kawano S, Yamano K (2011) BamE structure: the assembly of beta-barrel proteins in the outer membranes of bacteria and mitochondria. EMBO Rep. 10.1038/embor.2010.217 PMC3049440 21252940

[20] Heuck A, Schleiffer A, Clausen T (2011) Augmenting beta-augmentation: structural basis of how BamB binds BamA and may support folding of outer membrane proteins. J Mol Biol 406: 659–666. 21236263 10.1016/j.jmb.2011.01.002

[21] Kim KH, Paetzel M (2011) Crystal structure of Escherichia coli BamB, a lipoprotein component of the beta-barrel assembly machinery complex. J Mol Biol 406: 667–678. 21168416 10.1016/j.jmb.2010.12.020

[22] Knowles TJ, Browning DF, Jeeves M, Maderbocus R, Rajesh S, et al.. (2011) Structure and function of BamE within the outer membrane and the beta-barrel assembly machine. EMBO Rep. 10.1038/embor.2010.202 PMC3049429 21212804

[23] Noinaj N, Fairman JW, Buchanan SK (2011) The crystal structure of BamB suggests interactions with BamA and its role within the BAM complex. J Mol Biol 407: 248–260. 21277859 10.1016/j.jmb.2011.01.042 PMC3048904

[24] Sandoval CM, Baker SL, Jansen K, Metzner SI, Sousa MC (2011) Crystal Structure of BamD: An Essential Component of the beta-Barrel Assembly Machinery of Gram-Negative Bacteria. J Mol Biol 409: 348–357. 21463635 10.1016/j.jmb.2011.03.035 PMC3098899

[25] Kim KH, Aulakh S, Paetzel M (2011) Crystal structure of the {beta}-barrel assembly machinery BamCD complex. J Biol Chem. 10.1074/jbc.M111.298166 PMC3234736 21937441

[26] Vuong P, Bennion D, Mantei J, Frost D, Misra R (2008) Analysis of YfgL and YaeT interactions through bioinformatics, mutagenesis, and biochemistry. J Bacteriol 190: 1507–1517. 18165306 10.1128/JB.01477-07 PMC2258660

[27] Otwinowski Z, Minor W (1997) Processing of X-ray diffraction data collected in oscillation mode. Methods in Enzymology 276: 307–326. 10.1016/S0076-6879(97)76066-X 27754618

[28] Adams PD, Afonine PV, Bunkoczi G, Chen VB, Davis IW, et al (2010) PHENIX: a comprehensive Python-based system for macromolecular structure solution. Acta Crystallogr D Biol Crystallogr 66: 213–221. 20124702 10.1107/S0907444909052925 PMC2815670

[29] Emsley P, Cowtan K (2004) Coot: model-building tools for molecular graphics. Acta Crystallogr D Biol Crystallogr 60: 2126–2132. 15572765 10.1107/S0907444904019158

[30] Eddy SR (2009) A new generation of homology search tools based on probabilistic inference. Genome Inform 23: 205–211. 20180275

[31] Ashkenazy H, Erez E, Martz E, Pupko T, Ben-Tal N (2010) ConSurf 2010: calculating evolutionary conservation in sequence and structure of proteins and nucleic acids. Nucleic Acids Res 38 Suppl: W529–533 20478830 10.1093/nar/gkq399 PMC2896094

[32] Datsenko KA, Wanner BL (2000) One-step inactivation of chromosomal genes in Escherichia coli K-12 using PCR products. Proc Natl Acad Sci U S A 97: 6640–6645. 10829079 10.1073/pnas.120163297 PMC18686

[33] Lutz R, Bujard H (1997) Independent and tight regulation of transcriptional units in Escherichia coli via the LacR/O, the TetR/O and AraC/I1-I2 regulatory elements. Nucleic Acids Res 25: 1203–1210. 9092630 10.1093/nar/25.6.1203 PMC146584

[34] Tokuda H, Matsuyama S (2004) Sorting of lipoproteins to the outer membrane in E. coli. Biochim Biophys Acta 1693: 5–13. 15276320 10.1016/j.bbamcr.2004.02.005

[35] Ghosh M, Anthony C, Harlos K, Goodwin MG, Blake C (1995) The refined structure of the quinoprotein methanol dehydrogenase from Methylobacterium extorquens at 1.94 A. Structure. 3: 177–187. 10.1016/s0969-2126(01)00148-4 7735834

[36] Anthony C, Ghosh M (1998) The structure and function of the PQQ-containing quinoprotein dehydrogenases. Prog Biophys Mol Biol 69: 1–21. 9670773 10.1016/s0079-6107(97)00020-5

[37] Gruber J, Zawaira A, Saunders R, Barrett CP, Noble ME (2007) Computational analyses of the surface properties of protein-protein interfaces. Acta Crystallogr D Biol Crystallogr 63: 50–57. 17164526 10.1107/S0907444906046762 PMC2483497

[38] Ruiz N, Falcone B, Kahne D, Silhavy TJ (2005) Chemical conditionality: a genetic strategy to probe organelle assembly. Cell 121: 307–317. 15851036 10.1016/j.cell.2005.02.014

[39] Harrison SC (1996) Peptide-surface association: the case of PDZ and PTB domains. Cell 86: 341–343. 8756715 10.1016/s0092-8674(00)80105-1

[40] Gatsos X, Perry AJ, Anwari K, Dolezal P, Wolynec PP, et al (2008) Protein secretion and outer membrane assembly in Alphaproteobacteria. FEMS Microbiol Rev 32: 995–1009. 18759741 10.1111/j.1574-6976.2008.00130.x PMC2635482

[41] Richardson JS, Richardson DC (2002) Natural beta-sheet proteins use negative design to avoid edge-to-edge aggregation. Proc Natl Acad Sci U S A 99: 2754–2759. 11880627 10.1073/pnas.052706099 PMC122420

[42] Robert V, Volokhina EB, Senf F, Bos MP, Van Gelder P, et al (2006) Assembly factor Omp85 recognizes its outer membrane protein substrates by a species-specific C-terminal motif. PLoS Biol 4: e377. 17090219 10.1371/journal.pbio.0040377 PMC1634882

[43] Struyve M, Moons M, Tommassen J (1991) Carboxy-terminal phenylalanine is essential for the correct assembly of a bacterial outer membrane protein. J Mol Biol 218: 141–148. 1848301 10.1016/0022-2836(91)90880-f

[44] ter Haar E, Harrison SC, Kirchhausen T (2000) Peptide-in-groove interactions link target proteins to the beta-propeller of clathrin. Proc Natl Acad Sci U S A 97: 1096–1100. 10655490 10.1073/pnas.97.3.1096 PMC15533

[45] Morgunova E, Tuuttila A, Bergmann U, Tryggvason K (2002) Structural insight into the complex formation of latent matrix metalloproteinase 2 with tissue inhibitor of metalloproteinase 2. Proc Natl Acad Sci U S A 99: 7414–7419. 12032297 10.1073/pnas.102185399 PMC124245

[46] Anwari K, Poggio S, Perry A, Gatsos X, Ramarathinam SH, et al (2010) A modular BAM complex in the outer membrane of the alpha-proteobacterium Caulobacter crescentus. PLoS One 5: e8619. 20062535 10.1371/journal.pone.0008619 PMC2797634

[47] Webb C, Selkrig J, Perry A, Noinaj N, Buchanan SK, et al.. (2012) Dynamic Association of BAM Complex Modules Includes Surface Exposure of the Lipoprotein BamC. J Mol Biol. 10.1016/j.jmb.2012.05.035 PMC3433275 22683355

