# Supplementary figures and images for: Crystal Structure of BamB from Pseudomonas aeruginosa and Functional Evaluation of Its Conserved Structural Features
Source: PLoS One. 2012 Nov 26;7(11):e49749. doi: 10.1371/journal.pone.0049749 (PMC3506653; doi:10.1371/journal.pone.0049749)

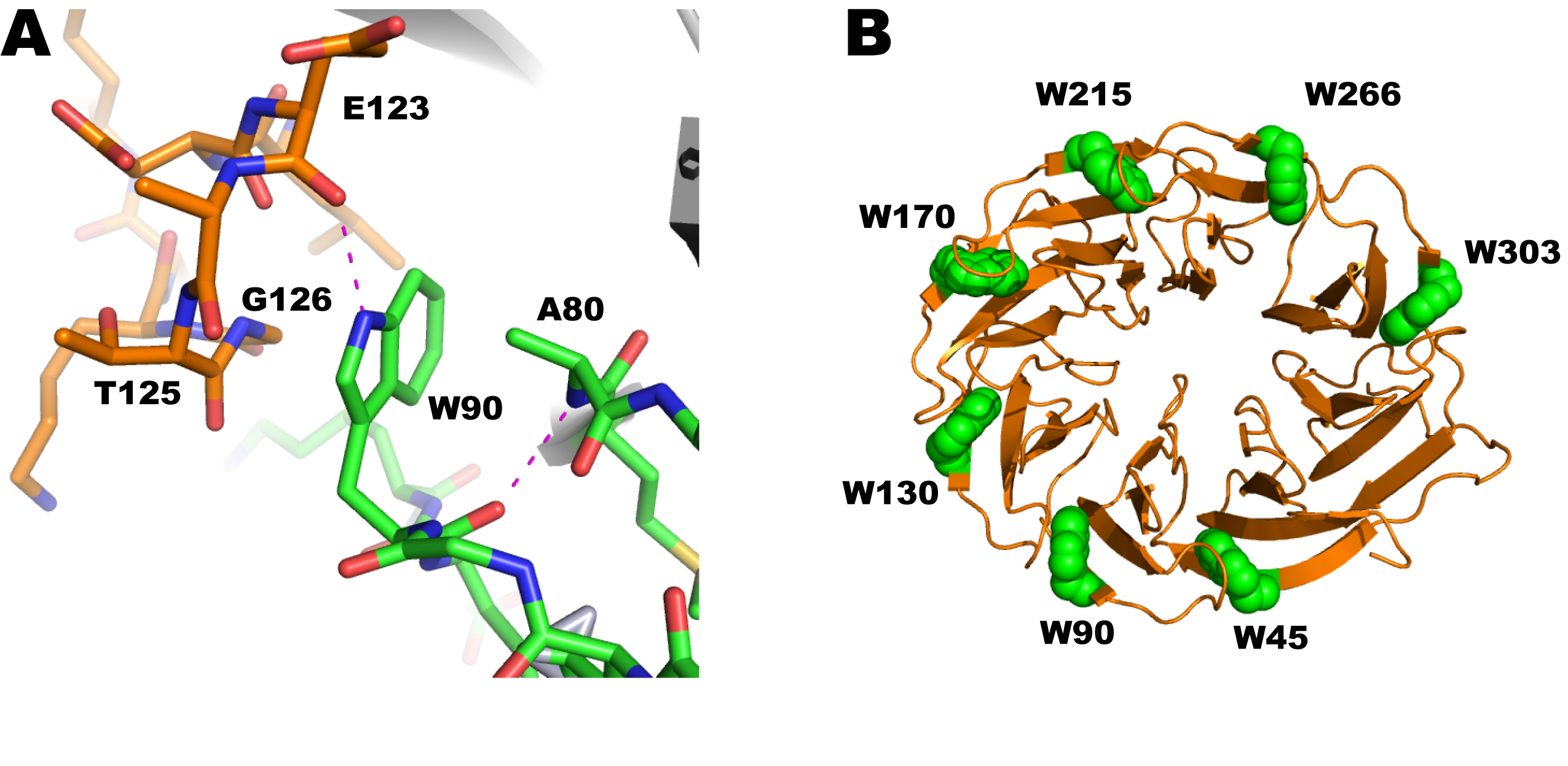

Supplement: Figure S1 — The Tryptophan Docking Motif (A) Close up of a Trp docking motif in BamB. The amide NH of A80 from the motif in one blade (green) hydrogen bonds with the carbonyl of W90 in the same motif. The side chain of W90 docks against the planar peptide bond between T125 and G126 in the motif on the next blade (orange). The indole NH of W90 also hydrogen bonds with the main chain carbonyl from E123 in the next blade. The tryptophan and the glycine are invariant residues and the interactions of the tryptophan with the main chain of both blades are the hallmark of the motif. For clarity, several side chains are not displayed. (B) Distribution of Trp docking motifs on BamB. The motifs stabilize seven of the eight blades in the BamB propeller forming a Trp girdle. (TIF) [file pone.0049749.s001.tif]

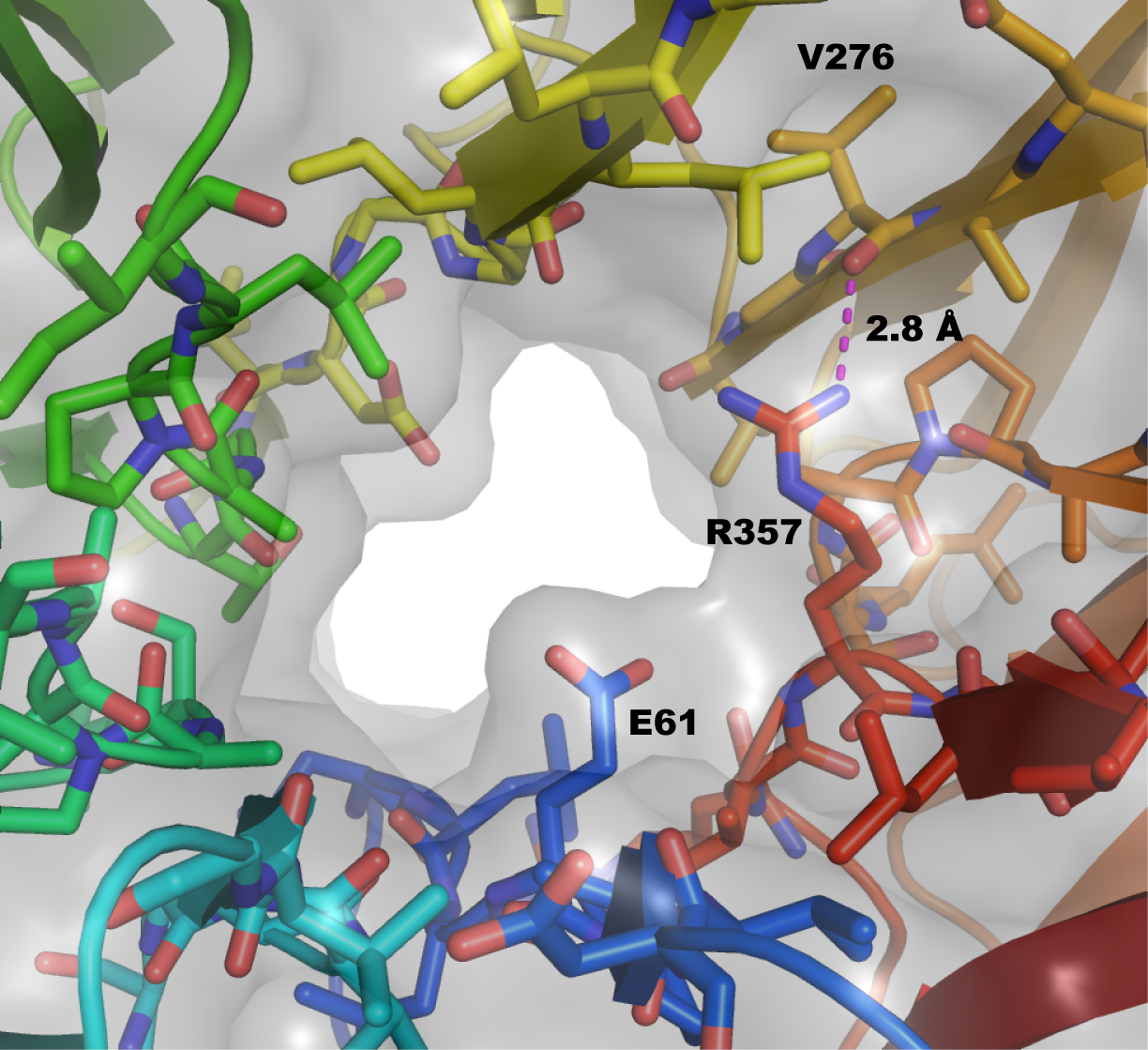

Supplement: Figure S2 — The Central Pore in BamB. A semitransparent surface representation of BamB, viewed down the central pore, reveals the packing of side chains lining the pore. E61 and R357 provide the main constrain to the volume of the pore. However, different rotamers could enlarge its capacity. In the crystal structure, the side chain of R357 hydrogen bonds the carbonyl of V276 in the A6 strand. (TIF) [file pone.0049749.s002.tif]

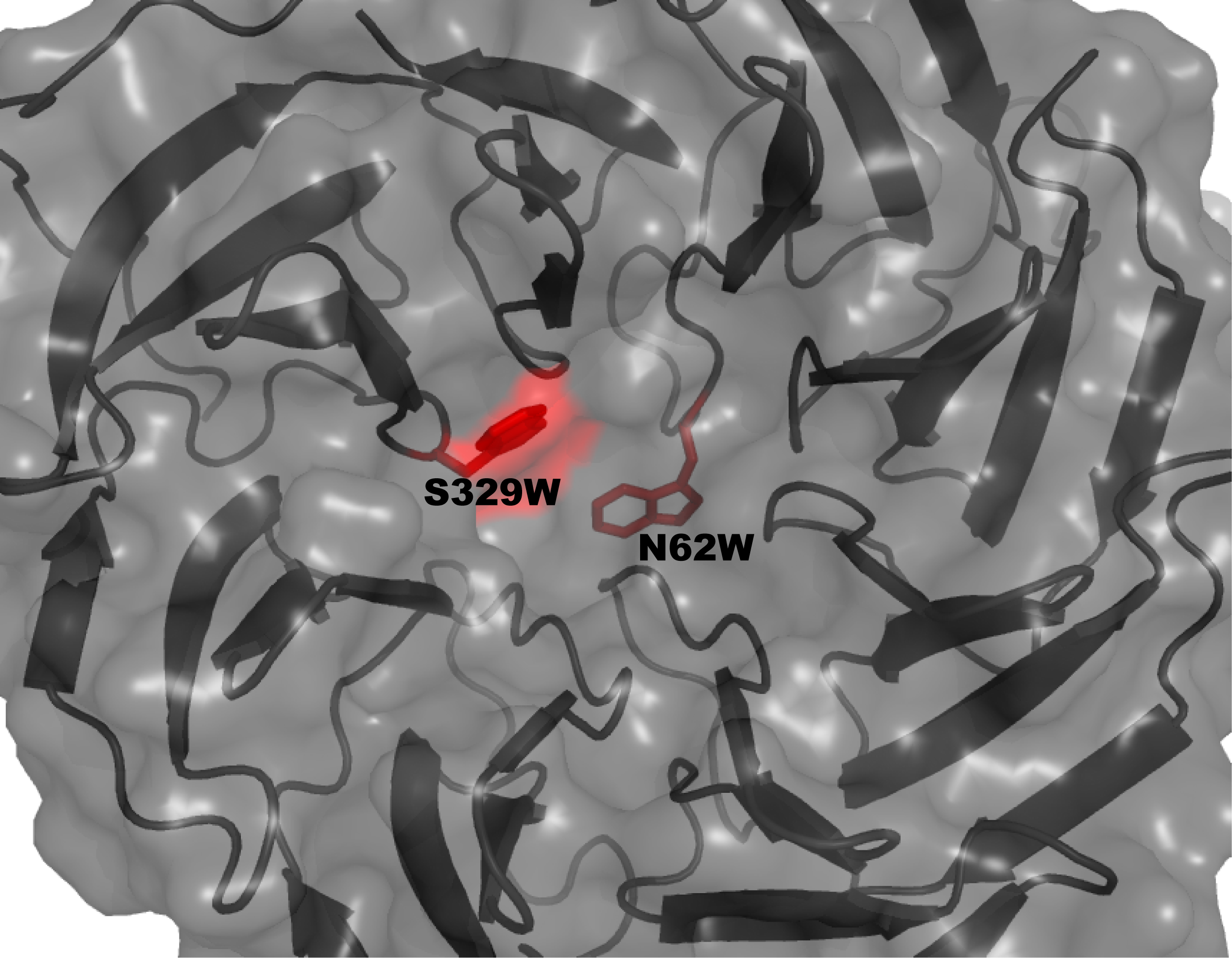

Supplement: Figure S3 — Mutations Blocking the Central Pore in BamB. Modeling of side chains in ecBamB N62W/S329W double mutant result in occlusion of the central pore in the protein. (TIF) [file pone.0049749.s003.tif]

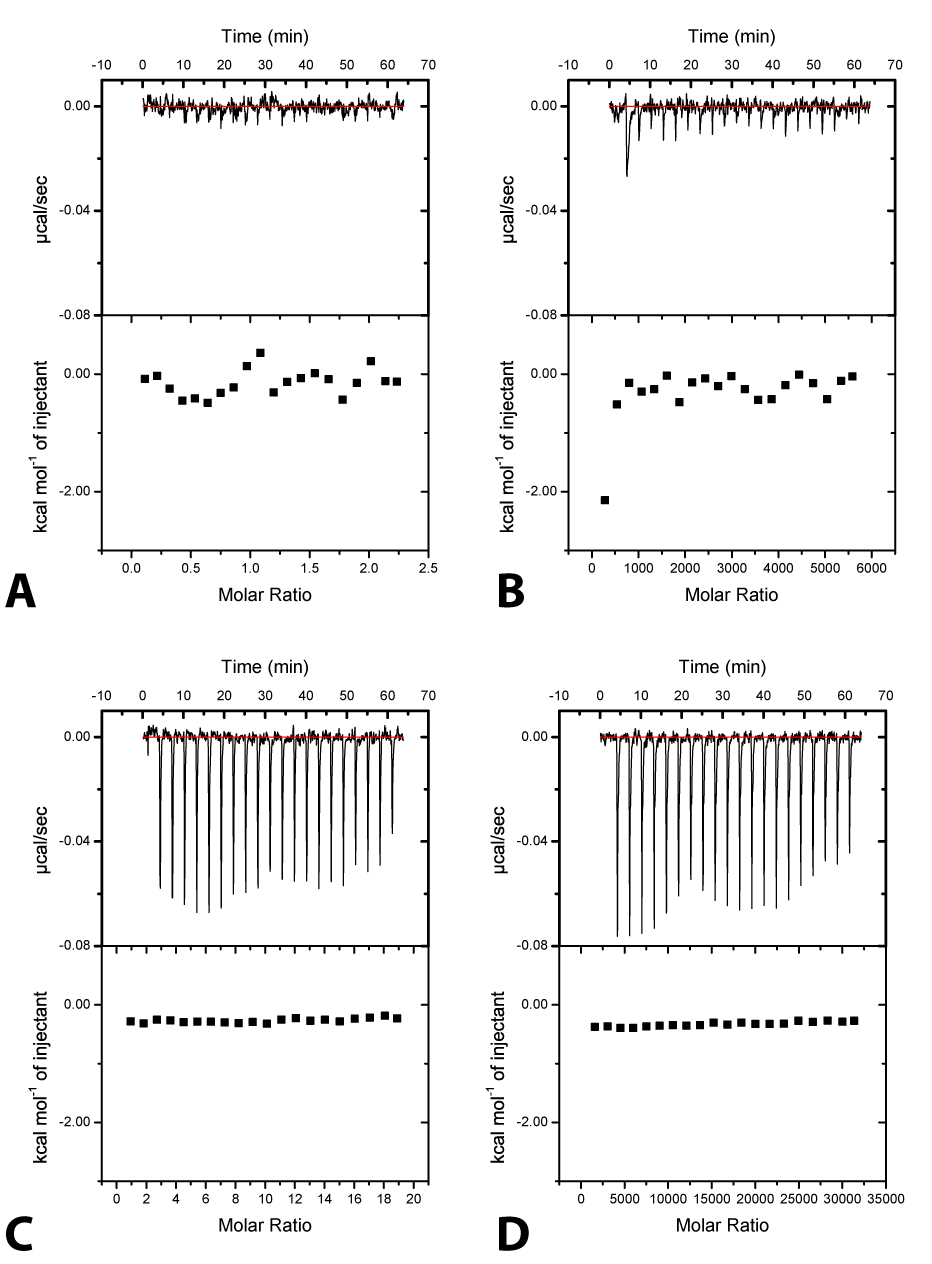

Supplement: Figure S4 — Representative ITC traces for the titration of β-barrel derived peptides on BamB. For each panel (A–D), the top plot represents the heat evolved after each injection (µCal/sec) vs time; while the bottom section shows the enthalpy (kcal/mol) as a function of peptide:ecBamB molar ratio (note: “the peptide into buffer titrations (panels B and D) show arbitrary numbers for “molar ratio” as no protein is present). The titration of 203 µM LamB peptide (sequence DFHGYARSGIGWT) on 25 µM ecBamB (A) is essentially the same as the titration of the same peptide solution into buffer (B). Similarly, titration of 1.5 mM BtuB peptide (sequence EYTLSGSYTF) on 17 µM ecBamB (C) was indistinguishable from the titration of the same peptide solution into buffer (D). We thus conclude that the observed signals are due to the heat of peptide dilution and no additional heat due to peptide binding to ecBamB is observed under these conditions. Titration of BtuB peptide was done at a large excess of peptide to account for the possibility of multiple binding sites. However, experiments carried out at peptide:BamB molar ratios in the range 1 to 3.5 were identical (data not shown), also indicating no detectable binding of the peptide to ecBamB. Isothermal Titration Calorimetry method details: Purified ecBamB was extensively dialyzed against buffer A (25 mM TrisHCl pH 8.0, 150 mM NaCl) prior to the ICT experiments. The peptide derived from the β-barrel of LamB: DFHGYARSGIGWT (>98% pure, synthesized by Creative Peptides), was dissolved in 50% DMSO and then subjected to buffer exchange on a PD-G10 column equilibrated in buffer A. Final peptide concentration was measured by UV absorbance at 280 nm using an extinction coefficient of 6,990 M−1 cm−1 (calculated from the amino acid sequence). The peptide derived from the C-terminal strand of BtuB: EYTLSGSYTF (>98% pure, synthesized by Anaspec) was soluble in buffer A but was also passed through a PVDF 0.1 µm filter equilibrated in buffer A. Final peptide [file pone.0049749.s004.tif]
