# Supplementary material for: Crystal Structure of BamB from Pseudomonas aeruginosa and Functional Evaluation of Its Conserved Structural Features
Source: PLoS One. 2012 Nov 26;7(11):e49749. doi: 10.1371/journal.pone.0049749 (PMC3506653; doi:10.1371/journal.pone.0049749)
Supplement: Table S1 — Primers used in the construction of plasmids. (DOC) [file pone.0049749.s005.doc]

| Plasmid name: | Description: | Primers used to construct plasmids: |
| --- | --- | --- |
| pMS906 | GFP | Digested and ligated between KpnI and XbaI sites. |
| pMS829 | ecBamB wild type | U666: 5’-GCCTCTAGATTAACGTGTAATAGAGTACACGGTTCC-3' |
|  |  | U666-1: 5'-GCCTCTAGATTAGTGATGATGATGATGATGACGTGTAATAGAGTA-3' |
| pMS907 | paBamB wild type | U668: 5'-TTAAGCGGCTGTAGCAGCAACAGCAAGAAGGAACTCC-3 |
|  |  | L668-1: 5'-GATGCCTCTAGATTAGTGATGATGATGATGATGCCCGGGGCG-3' |
|  |  | U667: 5'-TAATCTAGAGGCATCAAATAAAACGAAAGGCTCAG-3' |
|  |  | L671: 5'-ACAGCCGCTTAAAAGGGTAACGGAAAG-3' |
| pMS911 | N62W, S329W ecBamB | U688: 5'-GCAACTTCTATTCCTGGCTTCATCCGGCACTG-3' |
|  |  | L688: 5'-CAGTGCCGGATGAAGCCAGGAATAGAAGTTGC-3' |
|  |  | U689: 5'-GCAACTTCTATTCCTGGCTTCATCCGGCACTG-3' |
|  |  | L689: 5'-CAGTGCCGGATGAAGCCAGGAATAGAAGTTGC-3' |
| pMS912 | D288P ecBamB | U690: 5'-CTGGGTTCGGTGAATCCCTTCATCGTCGACG-3' |
|  |  | L690: 5'-CGTCGACGATGAAGGGATTCACCGAACCCAG-3' |
| pMS913 | F289P ecBamB | U691: 5'-GTTCGGTGAATGATCCCATCGTCGACGGCAATC-3' |
|  |  | L691: 5'-GATTGCCGTCGACGATGGGATCATTCACCGAAC-3' |
| pMS920 | missing extra loop ecBamB | U812: 5'-CTGGTCTGTCAGCCTGGCCGGGGCATTACTTTCTGGCGGTG-3' |
|  |  | L812: 5'-CACCGCCAGAAAGTAATGCCCCGGCCAGGCTGACAGACCAG-3' |
| pMS923 | D341A ecBamB | U767: 5'-GCAACCTGGTGGTCGGTGCCAGTGAAGGTTATCTGCA -3' |
|  |  | L767: 5'-GTGCAGATAACCTTCACTGGCACCGACCACCAGGTTGC-3' |
| pMS1001 | L35A, R325A, Q359A ecBamB | U867: 5'-GTAAAGATGTCCCCAGCACCAACCGTTGAAAAC-3' |
|  |  | L867: 5'-GTTTTCAACGGTTGGTGCTGGGGACATCTTTAC-3' |
|  |  | U766: 5'-CAAAGCGATCTGCTGCATGCCCTGCTGACTTCTCC-3' |
|  |  | L766: 5'-GGAGAAGTCAGCAGGGCATGCAGCAGATCGCTTTG-3' |
|  |  | U866: 5'-GTCGTTTCGTTGCCGCACAAAAAGTTGATAG-3' |
|  |  | L866: 5'-CTATCAACTTTTTGTGCGGCAACGAAACGAC-3' |
